# The Influence of Clinical Factors and Genetic Variants of *COL1A1* and *TNFRSF11B* on Bone Mineral Density in Postmenopausal Women

**DOI:** 10.3390/ijms26188894

**Published:** 2025-09-12

**Authors:** Katarzyna Kotrych, Maciej Wojtuń, Aleksandra Górska, Anna Bogacz, Michał Soczawa, Izabela Uzar, Jarosław Gorący, Maciej Brązert, Bogusław Czerny, Adam Kamiński

**Affiliations:** 1 Department of General and Dental Radiology, Pomeranian Medical University in Szczecin, al. Powstańców Wielkopolskich 72, 70-111 Szczecin, Poland; kotrych1@gmail.com (K.K.); maciej.wojtun@pum.edu.pl (M.W.); 2 Department of Stem Cells and Regenerative Medicine, Institute of Natural Fibres and Medicinal Plants, Kolejowa 2, 62-064 Plewiska, Poland; aleksandra.gorska@iwnirz.pl (A.G.); bczerny@wp.pl (B.C.); 3 Department of Cancer Immunology, Poznan University of Medical Sciences, Rokietnicka 8, 60-806 Poznan, Poland; 4 Department and Clinic of Urology and Urological Oncology, Pomeranian Medical University in Szczecin, al. Powstańców Wielkopolskich 72, 70-111 Szczecin, Poland; michal.soczawa@pum.edu.pl; 5 Department of Pharmacology and Pharmacoeconomics, Pomeranian Medical University in Szczecin, 71-230 Szczecin, Poland; uzari@wp.pl; 6 Independent Laboratory of Invasive Cardiology, Pomeranian Medical University in Szczecin, 70-111 Szczecin, Poland; jaroslaw.goracy@pum.edu.pl; 7 Department of Infertility Diagnostics and Treatment, Poznan University of Medical Sciences, Polna 33, 60-535 Poznan, Poland; maciejbrazert@ump.edu.pl; 8 Department of Children Orthopedics and Musculosceletal Oncology, Pomeranian Medical University in Szczecin, Unii Lubelskiej 1, 71-252 Szczecin, Poland; emluc@wp.pl

**Keywords:** osteoporosis, polymorphism, *COL1A1*, *TNFRSF11B*, BMD, postmenopausal women

## Abstract

Osteoporosis is a chronic metabolic disease characterised by reduced bone mineral density (BMD) and increased susceptibility to fractures. Its development is influenced by both environmental and genetic factors that regulate bone metabolism. Among the genes involved in bone metabolism, *COL1A1* and *TNFRSF11B* (OPG) are particularly important. The *COL1A1* gene encodes the alpha-1 chain of type I collagen, a major component of the bone matrix, and plays a key role in maintaining bone mechanical strength. The *TNFRSF11B* gene encodes osteoprotegerin (OPG), a protein that inhibits bone resorption by binding the RANKL ligand and blocking osteoclast activation. Therefore, the aim of this study was to determine the association between the rs1107946 and rs1800012 polymorphisms of the *COL1A1* gene and the rs2073617 polymorphism of the *TNFRSF11B* (OPG) gene and bone mineral density in postmenopausal women. The study included 590 postmenopausal women: 350 healthy controls, 105 with osteopenia, and 135 with osteoporosis. Genotyping was performed using real-time PCR and LightSNiP probes. Associations between genetic variables and BMD were assessed, taking into account environmental factors (BMI, smoking). The presence of the T allele of the rs1800012 variant was initially associated with lower BMD and an increased risk of osteopenia, but this association lost significance after adjustment for BMI and smoking. For rs1107946 and rs2073617,no statistically significant associations were observed. These findings suggest that the studied SNPs have, at most, modest effects on BMD, with environmental influences playing a stronger role. Further research in larger and more diverse cohorts, including FRAX-based risk estimation, is warranted.

## 1. Introduction

Osteoporosis is a chronic, metabolic disease of the skeletal system, the development of which depends on the interaction of genetic and environmental factors [1]. It is characterised by decreased bone mass, deterioration of bone microarchitecture, and increased fragility, resulting in a higher fracture risk [2,3]. It is estimated that one in three women and one in five men over the age of 50 will experience osteoporotic fractures [1,4]. This disease particularly affects women in the perimenopausal period due to hormonal changes that contribute to bone metabolism disorders [3]. Oestrogens play a crucial role in bone remodelling by inhibiting osteoblast apoptosis and promoting osteoclast apoptosis [5]. The decline in oestrogen levels during menopause leads to increased osteoblast apoptosis and increased osteoclast activity, accelerating bone resorption, bone loss, and ultimately fracture risk.

Genetic factors play a significant role in the pathogenesis of osteoporosis by influencing peak bone mass (PBM) and bone mineral density (BMD). Numerous genes are involved in maintaining bone homeostasis, including those encoding type I collagen (*COL1A1*, *COL1A2*), osteoprotegerin (*OPG*/*TNFRSF11B*), the vitamin D receptor (*VDR*), and the oestrogen receptor (*ER*) [6,7,8,9]. The *COL1A1* and *COL1A2* genes, encoding type I collagen—the main component of the organic matrix of bone—play an important role in the physiology and pathology of the skeletal system. *COL1A1* is located on chromosome 17 (locus 17q21.33) and consists of 51 exons, while *COL1A2* is located on chromosome 7 (locus 7q21.3-q22) and consists of 52 exons [10,11]. Mutations in *COL1A1* are associated with osteogenesis imperfecta, and its polymorphisms can affect bone density and the development of osteogenesis imperfecta in 85–90% of cases [5,12].

Osteoprotegerin, a glycoprotein from the TNF receptor family, plays a key role in regulating bone resorption by inhibiting osteoclast activation. It is encoded by the *TNFRSF11B* (*OPG*) gene, located on chromosome 8 (region 8q24.12), and comprises five exons. OPG blocks the action of the RANKL factor, necessary for the activation of osteoclasts [13,14]. Several polymorphisms of this gene, including rs3134069, rs3102735, rs2073617, and rs2073618, have been repeatedly analysed for their association with the risk of osteoporosis [15].

The aim of the present study was to examine the association between the rs1107946 and rs1800012 polymorphisms of the *COL1A1* gene as well as the rs2073617 polymorphism of the *TNFRSF11B* gene, and bone mineral density in postmenopausal women. These SNPs were selected because previous studies demonstrated their potential role in BMD and fracture risk. The rs1800012 polymorphism (*COL1A1*, Sp1 site) alters Sp1 transcription factor binding, leading to changes in collagen structure and an increased fracture risk [16,17]. The rs1107946 variant (*COL1A1* promoter region) may affect transcriptional activity and has been associated with differences in BMD in some populations [18,19]. The rs2073617 polymorphism (*TNFRSF11B* promoter region) influences osteoprotegerin expression and has been linked to BMD variability and osteoporosis risk in meta-analyses [15,20,21,22]. Building on this evidence, we focused on these three variants to evaluate their relevance in a Polish postmenopausal cohort. Understanding the role of these relationships may be important for the development of more effective diagnostic and therapeutic strategies aimed at early identification and treatment of patients at increased risk.

## 2. Results

### 2.1. Clinical Data Analysis

Table 1 presents the general clinical characteristics of the patients included in the study. The mean age was 54.83 ± 7.59 years, the youngest participant was 30, and the oldest was 78 years. The mean birth weight was 3284.63 ± 504.77 g, and the current weight was 66.24 ± 11.57 kg (range 41 to 114 kg). The minimum body mass index was 17.10 kg/m^2^, and the maximum was 43.43 kg/m^2^ (mean 25.16 ± 4.18 kg/m^2^). A total of 251 women (42.54%) were overweight or obese. The study participants had their first period on average at 13 years (range 9–17 years), and their last at 48 years (range 30–60). On average, women were 5 years postmenopausal (range: 1–26 years) and had two pregnancies (range: 0–7) during their 36 reproductive years (range: 17–48). More than a quarter of respondents (26.44%) reported that they currently smoke or have smoked cigarettes for at least 5 years of their life.

In analysing the correlations between BMD and clinical data in the study group, statistically significant positive correlations were found with birth weight (rho = 0.32; 95%CI 0.24–0.39; *p* < 0.001) and current body weight (rho = 0.30; 95%CI 0.23–0.38; *p* < 0.001), BMI (rho = 0.29; 95%CI 0.21–0.37; *p* < 0.001), and the number of pregnancies (rho = 0.18; 95%CI 0.09–0.25; *p* < 0.001).

Patients who obtained a T-score above −1 in the densitometric examination, indicating normal bone mineral density, were classified into the control group (N = 350), while those with a T-score ≤ −1 were classified into the group with reduced bone mineral density (N = 240). Mean values for clinical variables were compared in these two groups, and the results are presented in Table 2. The groups did not differ statistically significantly in age (54.6 ± 7.2 vs. 55.2 ± 8.1, *p* = 0.293), time of first and last menstruation, number of reproductive years, and previous pregnancies. Statistically significantly lower means were found in the group with reduced BMD for birth weight (median: 3095 g vs. 3410 g, *p* < 0.001) and current body weight (62.7 ± 9.9 kg vs. 68.7 ± 12.0 kg, *p* < 0.001), height (161.1 ± 5.1 cm vs. 162.9 ± 5.8 cm, *p* < 0.001), and BMI (24.1 ± 3.4 vs. 25.9 ± 4.5 kg/m^2^, *p* < 0.001). In the group with normal bone mineral density values, there were almost 10% more women who were overweight or obese (46.6% vs. 36.7%, *p* = 0.021). Patients in the lower-T-score group were more years postmenopausal (*p* = 0.005) and more likely to smoke cigarettes (*p* = 0.036) (Table 2).

Descriptive statistics of bone mineral density parameters of the lumbar spine L2–L4 using DXA in the control, osteopenia, and osteoporosis groups are summarised in Table 3. Median BMD was 1.179 g/cm^2^ [IQR: 1.121;1.235, range 1.08 to 1.47] in the control group, 0.972 g/cm^2^ [IQR: 0.938;1.032, range 0.90 to 1.07] in osteopenia, and 0.822 g/cm^2^ [IQR: 0.774; 0.875, range 0.63 to 0.90] in osteoporosis.

### 2.2. Association Analysis of COL1A1 Gene Polymorphic Variants with Reduced Bone Mineral Density

Assuming an alpha value of 0.05 to detect an odds ratio of 2.0 in a case—control study with 590 patients, including 240 cases and 350 controls (case prevalence 40.7%), and MAF values of 14.43% (rs1800012), 18.29% (rs1107946), and 46.00% (rs2073617), as observed for the controls, the dominant and additive models achieved power exceeding 80%.

This study analysed two polymorphic variants of the *COL1A1* gene: rs1800012 (NG_007400.1:g.6252G>T) and rs1107946 (NG_007400.1:g.3011T>G). In the entire study group, the frequency of rs1800012 genotypes was GG 416 (70.51%), GT 158 (26.78%), and TT 16 (2.71%). For the rs1107946 variant, the homozygous GG genotype (66.78%) was obtained in 394 women, the heterozygous GT genotype (30.17%) in 178 women, and the heterozygous GT genotype (30.17%) in 18 individuals with the TT genotype (3.05%). The obtained genotype frequencies allowed us to calculate allele frequencies and their concordance with the expected frequencies according to the Hardy—Weinberg law. Alleles less frequently occurring in the population (minor allele) occurred for the rs1800012 polymorphism in 190 women (16.10%) and for the rs1107946 polymorphism in 214 individuals (18.14%). Both *COL1A1* gene variants met the assumptions of Hardy—Weinberg equilibrium (*p* = 0.879 for rs1800012 and *p* = 0.782 for rs1107946). A polymorphic variant of the *TNFRSF11B* (tumour necrosis factor receptor superfamily member 11b) gene, formerly known as *OPG* (osteoprotegerin), a member of the tumour necrosis factor receptor family, was also examined. The rs2073617 variant (NG_012202.1:g.5101C>T), located in the 5′ untranslated region (5′UTR) in the gene promoter region, was selected for analysis. In the study group, the TT genotype occurred in 172 women (29.15%), TC heterozygote in 277 (46.95%), and CC homozygote in 141 (23.90%). The allele frequency for T was 52.63%, while for C it was 47.37%, consistent with the Hardy—Weinberg law (*p* = 0.161). Table 4 and Table 5 present the results of the association analysis for the tested *COL1A1* and *TNFRSF11B* gene variants for the crude and adjusted models. A group of 240 patients with a T-score ≤ −1 was compared with 350 individuals with normal BMD (T-score above −1). In the crude logistic regression model, a statistically significant value was obtained for rs1800012 in the dominant model. Genotypes containing at least one mutant T allele (GT and TT) were more frequent in patients with reduced BMD (34.2% vs. 26.3% in controls, OR = 1.46, 95%CI: 1.02–2.08, *p* = 0.040, AIC = 797.1) (Table 4).

In the model adjusted for BMI and smoking, the dominant model was the best, but a statistically significant value was not obtained (OR = 1.33, 95%CI: 0.92–1.92, *p* = 0.136, AIC = 767.9) (Table 5). In the case of the rs1107946 and rs2073617 polymorphisms, no significant associations with reduced BMD were observed, regardless of the genetic model considered, both in the crude model and in the model adjusted for BMI and smoking.

The allele frequencies of both studied SNVs of the *COL1A1* gene are presented in Table 6. The T allele for rs1800012 was more frequent in women with reduced BMD compared to the control group (18.54% vs. 14.43%, OR = 1.35, 95%CI: 0.99–1.84; *p* = 0.060). For the rs1107946 variant, the minor allele T was present in 18.29% of controls and in 17.92% of women with a T-score less than or equal to −1 (OR = 0.98, 95%CI: 0.72–1.32; *p* = 0.872). In the analysed rs2073617 polymorphism of the *TNFRSF11B* gene, a more frequent occurrence of the C allele was observed in women with reduced BMD compared to patients with normal bone mineral density (49.38% vs. 46.00%, OR = 1.14, 95%CI: 0.91–1.44, *p* = 0.254).

### 2.3. Association Analysis of Polymorphic Variants of the COL1A1 Gene in Groups with Osteopenia and Osteoporosis

Table S1 presents the results of the association of the studied variants with the occurrence of osteopenia compared to the control group. In the case of analysis of the rs1800012 *COL1A1* polymorphism in the dominant model, the odds ratio was 1.59 (95%CI: 1.00–2.53), which means that the presence of at least one T allele (TT or GT) increases the risk of osteopenia by 59% (*p* = 0.052). After adjustment for BMI and cigarette smoking, the OR = 1.46 (95%CI: 0.91–2.35; *p* = 0.118). The best model was the log-additive one, which was statistically significant in the crude model (*p* = 0.044). For the second SNP of the *COL1A1* gene, no statistically significant associations were obtained. There was also no statistically significant association of the rs2073617 variant of the *TNFRSF11B* gene with the risk of osteopenia in the codominant model (*p* = 0.393); after adjustment for BMI and smoking, it was *p* = 0.277. After adjustment for BMI and smoking, the presence of genotypes containing the C allele (CC and TC) reduced the risk of osteopenia by 29% compared to individuals with the homozygous TT genotype (dominant model OR = 0.71; 95%CI: 0.44–1.13; *p* = 0.155) (Table S1).

Associations of the studied *COL1A1* and *TNFRSF11B* gene variants with the occurrence of osteoporosis compared to the control group are presented in Table 7. For both *COL1A1* polymorphic variants, no statistically significant relationships were obtained in any of the genetic models. Analysis of the rs2073617 variant of the *TNFRSF11B* gene with the risk of osteoporosis indicates that in the adjusted model, the heterozygous TC genotype increases the risk by 30%, and the CC genotype by 78%, compared to the homozygous TT genotype (*p* = 0.155). The best model was the log-additive one, for which a statistically significant value was obtained in the crude model (*p* = 0.026), while after adjustment, *p* = 0.054 (Table 7).

### 2.4. Associations of the rs1800012 Variant of the COL1A1 Gene with Clinical and Densitometric Data

In comparing the means for clinical data of patients divided into three subgroups depending on their rs1800012 variant genotypes, statistically significant differences were observed for current body weight and BMI. Women with the homozygous GG genotype had a higher current body weight of 67.3 ± 11.6 kg compared to those with the heterozygous genotype (64.1 ± 11.1 kg) and the homozygous TT genotype (60.8 ± 12.6 kg) (*p* = 0.002). Nearly half of those (47.1%) with the GG genotype had a BMI greater than or equal to 25 (mean 25.5 ± 4.2 kg/m^2^). In individuals with genotypes containing the T allele, overweight or obesity was observed in 33.5% of GT heterozygotes (mean 24.4 ± 4.0 kg/m^2^) and in 12.5% of TT homozygotes (mean 22.9 ± 4.5 kg/m^2^). The remaining analysed variables did not differ statistically significantly between groups (Table S2). In comparing clinical parameters between alleles of this polymorphism, statistically significant differences were also observed in women’s current body weight and BMI. The mean body weight of the patients was 66.8 ± 11.5 kg for the G allele and 63.5 ± 11.4 kg for the T allele (*p* < 0.001). For BMI, the mean values were 25.4 ± 4.2 kg/m^2^ for the G allele and 24.1 ± 4.1 kg/m^2^ for the T allele (*p* < 0.001). The G allele was associated with an almost 15% increased risk of being overweight or obese compared with the T allele (44.9% vs. 30.0%, *p* < 0.001) (Table S2).

We also examined the relationship between current body weight and BMI observed for the entire study group based on T-scores obtained in the densitometric examination. The most interesting results were observed in the groups of women with normal bone mineral density (control) and those with osteopenia. The mean values for body weight and BMI in women in the control, osteopenia, and osteoporosis groups, divided by genotypes of the rs1800012 variant of the *COL1A1* gene, are summarised in Table S3.

The T allele of the rs1800012 variant of the *COL1A1* gene was associated with lower current body weight (66.2 ± 12.5 kg vs. 69.1 ± 11.9 kg for G, *p* = 0.026) and lower BMI (24.9 ± 46 vs. 26.1 ± 4.5 kg/m^2^ for G, *p* = 0.013) in the control group. In the osteopenia group, the mean body weight was 66.5 ± 11.2 kg for the G allele vs. 61.2 ± 9.7 for T, with *p* = 0.005. The mean BMI in this group was 25.1 ± 4.0 kg/m^2^ for G and 23.2 ± 3.7 kg/m^2^ for T, with *p* = 0.006. In the group of women with osteoporosis, no statistically significant differences were observed for mean body weight (60.8 kg ± 8.3 kg vs. 59.8 kg ± 8.3 kg, *p* = 0.435) and BMI (23.6 kg ± 2.6 kg vs. 23.4 kg ± 2.8 kg, *p* = 0.553) between the G and T alleles of the rs1800012 variant. When analysing densitometric variables by rs1800012 genotype, no statistically significant differences were observed, and the results are presented in Table S4.

### 2.5. Associations of the rs1107946 Variant of the COL1A1 Gene with Clinical and Densitometric Data

For the rs1107946 variant of the *COL1A1* gene, the average clinical data in the three genotype groups did not show any statistically significant differences. However, similarly to the rs1800012 variant, the mean body weight of patients with the homozygous GG genotype was the highest, at 66.7 ± 11.8 kg, for the heterozygous GT genotype, it was 65.6 ± 11.0 kg, and it was the lowest in women with the genotype containing both mutant alleles, at62.3 ± 12.6 kg (*p* = 0.195) (Table S5). Also, after stratifying the study group into control, osteopenia, and osteoporosis, no statistically significant relationships were observed. Analysis of densitometric data by genotype for this polymorphism did not reveal any statistically significant differences, and the results are presented in Table S6.

### 2.6. Associations of the rs2073617 Variant of the TNFRSF11B Gene with Clinical and Densitometric Data

Analysis of clinical data from all studied women, divided into three subgroups based on their genotypes for the rs2073617 variant of the *TNFRSF11B* gene, revealed statistically significant differences between median birth weights. Women with the homozygous TT genotype had the lowest weight, at 3260 g, those with the heterozygous TC genotype had a weight of 3300 g, and those with the homozygous CC had a weight of 3360 g (*p* = 0.009). The relationship between current body weight and BMI was reversed, with the highest for the TTgenotype and the lowest for the CCgenotype (*p* = 0.053 for body weight and *p* = 0.047 for BMI) (Table S7). Median birth weights for individual genotypes were then compared in the groups of women with normal BMD, osteopenia, and osteoporosis. In each group, the median birth weights of patients with the TT genotype were the lowest, and they were highest for the CC genotype. The differences between medians were statistically significant in the control groups with normal BMD (*p* = 0.023) and in osteopenia (*p* = 0.013) (Table S8).

The medians obtained for densitometric variables after stratifying by the TT, TC, and CC genotypes of the rs2073617 variant did not differ statistically significantly. However, higher medians were observed for densitometric parameters in comparing the patients’ bone density results with the mean bone density of healthy young adults in women with the TT genotype. The T-score for young adults was 94% for the TT genotype, 93% for TC heterozygotes, and 91% for the CC genotype (*p* = 0.081). The T-score calculated based on this parameter was also highest for individuals with the TT genotype (median −0.8 [IQR: −1.9; 0.2]) and lowest for the CC genotype (median −0.9 [IQR: −2.6; −0.2]), *p* = 0.091). The results obtained for bone mineral density parameters between genotypes of the rs2073617 variant of the *TNFRSF11B* gene are summarised in Table S9.

## 3. Discussion

Hormonal changes occurring in women during menopause have a significant impact on metabolic and bone health. One of the most serious complications of this period is the development of osteoporosis, the pathogenesis of which is primarily related to oestrogen deficiency. This deficiency leads to an imbalance between bone formation and resorption, resulting in a decrease in bone mineral density (BMD). Genetic factors, lifestyle, body weight, and comorbidities also play a significant role in the course and rate of these changes. According to the literature, genetic factors account for 60–80% of the variability in BMD [8].

Increasing attention is currently being paid to single-nucleotide polymorphisms (SNPs) that may influence bone metabolism. Among the analysed genes, the most significant are *COL1A1*, *ESR1*, *VDR*, *TNFRSF11B*, and *RANKL* [8]. The *COL1A1* gene, encoding the α1 chain of type I collagen, is crucial for maintaining the structural integrity of bone tissue. Polymorphisms of this gene, especially rs1800012 (Sp1) and rs1107946, may influence collagen expression and its physicochemical properties [18,23].

The rs1800012 polymorphism of the *COL1A1* gene, located in the Sp1 transcription factor binding site in intron 1, may disturb the α1:α2 chain ratio and affect the structural properties of collagen [22]. The T allele binds Sp1with higher affinity, leading to α1overexpression and imbalancedα1:α2. This can result in less stable homotrimers, weakening bone and increasing fracture susceptibility independently of BMD [16].

Our study demonstrated a significant association between the presence of the T allele (GT/TT) and lower bone mineral density. The frequency of these genotypes was higher in the group with reduced BMD (34.2% vs. 26.3%), and the additive model indicated a significant trend of increasing risk with increasing T allele number (OR = 1.50; *p* = 0.044). This effect lost significance after accounting for environmental variables such as BMI and smoking (OR = 1.33; *p* = 0.136), which may suggest that the genetic influence of rs1800012 is modified by environmental factors. Similar results have been reported in numerous meta-analyses and cohort studies, which confirmed the association of the T allele (also known as the “s” allele at the Sp1 binding site) with reduced BMD and higher fracture risk in European populations [17,19,23,24,25]. However, some studies, especially in populations with a smaller number of T allele carriers, did not confirm these relationships [26].

A subgroup analysis of women with osteopenia showed a 59% increased risk of the disease in the presence of the T allele (GT/TT) (OR = 1.59; *p* = 0.052), and the additive model achieved statistical significance (*p* = 0.044). After adjustment for BMI and smoking, this association lost significance (*p* = 0.118), which may indicate a masking effect of these factors. Similar results were obtained in the study by Singh et al. (N = 349), where the GT genotype was associated with a 2.76-fold higher risk of osteopenia [27].

In women with osteoporosis, rs1800012 was not significantly associated with BMD or fracture risk. This suggests that its role may be limited to earlier stages of the disease. These results are consistent with numerous reports, including a study of a Polish cohort (N = 311 women with osteoporosis), which found no association between the Sp1 genotype and BMD or fracture risk (*p* = 0.613) [26]. Such results were also reported in the large European GENOMOS cohort (>20,000 individuals), where the effect of the T (“s”) allele on BMD was only evident in the oldest participants (>80 years) [28]. These results suggest that the genetic effect of rs1800012 may be attenuated in advanced osteoporosis, where age, hormonal status, and physical activity play a more significant role. The study by Singh et al. (N = 349) revealed a strong risk of osteoporosis (OR 3.43, 95% CI 1.40–9, *p* < 0.004; 3-fold) [27]. The literature supports similar observations: a meta-analysis including 30 studies (N = 2943 patients vs. 4724 controls) showed a significant increase in the risk of osteoporosis and fractures in all genetic models, dominant, allelic, and heterozygous, with a clear effect also in the European subpopulation (dominant model significant, *p* < 0.001) [17].

Furthermore, T allele carriers were observed to have lower body weight and BMI (*p* < 0.001). In our study, 30% of women with this allele had a BMI ≥ 25, compared to 44.9% in G allele carriers, which may indicate an indirect, protective effect of the G allele via body weight. Although the mechanism of this phenomenon is unclear, it is possible that changes in collagen structure influence adipose tissue metabolism or distribution. These results support previous reports suggesting a possible indirect effect of the rs1800012 polymorphism on body composition [16].

The rs1107946 variant, located in the promoter region of the *COL1A1* gene, potentially affects the level of gene transcription and its expression in various tissues [18]. The full analysis revealed no significant differences in genotype frequencies between the groups with reduced and normal BMD. Subgroup analysis (women with osteopenia) also failed to reveal significant associations. These results are consistent with some studies, although the literature is not conclusive—some reports indicate an association of the T allele with higher BMD [18], while others do not confirm this [19].

No significant associations with the occurrence of osteoporosis were observed. In the clinical analysis, women with the GG genotype had a slightly higher BMI than GT heterozygotes (mean 25.3 vs. 25.0), which may suggest an indirect effect of this variant on body composition and mechanical loading of the skeleton, which is confirmed by previous reports [29].

Analysis of the rs2073617 variant of the *TNFRSF11B* gene, encoding osteoprotegerin, revealed no significant genotypic differences between the groups with reduced and normal BMD. In the dominant model, a reduced risk of osteopenia was observed with the presence of the C allele (OR = 0.71; *p* = 0.155), but this association did not reach significance. These results are consistent with previous reports [15,20].

Additionally, analysis of the *TNFRSF11B* rs2073617 variant did not demonstrate statistical significance in the risk of osteoporosis. However, in the additive model, a trend toward an increased risk of osteoporosis was observed with the C allele (crude model: *p* = 0.026; adjusted model: *p* = 0.054). Although this observation is not conclusive, it may indicate a small, biologically significant effect of this variant, and further studies in larger cohorts are needed. It is worth noting that the study found an association of rs2073617 with birth weight and bone characteristics in adulthood, suggesting a possible developmental role for this polymorphism. The research results are not clear—there are several reports suggesting the influence of rs2073617 on the risk of developing osteoporosis [21,22] as well as several that do not confirm the significance [20].

Moreover, the study demonstrated statistically significant positive correlations between BMD and three clinical variables: birth weight, current body weight, and BMI. The Spearman correlation coefficients of rho = 0.32 for birth weight, rho = 0.30 for current body weight, and rho = 0.29 for BMI indicate moderate but significant monotonic relationships. These results suggest that individuals with higher birth weight and higher BMI tend to have higher bone density in adulthood. The observation that birth weight positively correlates with BMD in adulthood may indicate the influence of prenatal conditions on skeletal development. Lower birth weight may reflect a suboptimal intrauterine environment (e.g., nutrient deficiencies or foetal growth restriction), which can lead to impaired achievement of peak bone mass and an increased risk of osteoporosis later in life. The results also indicate that higher body weight and BMI are associated with higher bone density. This may be due to both greater mechanical loading on the skeleton (which stimulates its remodelling) and hormonal influences (e.g., higher oestrogen concentrations in adipose tissue in women). However, it is worth noting that excess body weight can be a risk factor for other health problems, so potential “benefits” for bone should be considered with caution. The results confirm that both prenatal factors (birth weight) and ongoing factors (BMI, body weight) have a significant impact on bone health in adulthood. They emphasise the importance of early osteoporosis prevention, encompassing not only a healthy lifestyle in adulthood but also actions supporting the proper development of the foetus and child. The results may have significant implications for public health strategies, particularly in the context of an ageing population and the growing importance of skeletal diseases.

### Strengths and Limitations

Our study has several strengths, including a relatively large sample size, careful exclusion of secondary osteoporosis, and detailed clinical data collection. Nonetheless, certain limitations should be acknowledged.

One limitation is that the proportion of women with early menopause (≤45 years) was higher than usually reported in the general population. This may partly reflect the characteristics of the regional cohort, where factors such as smoking prevalence, occupational exposures, and nutritional habits could contribute to earlier ovarian ageing. Another explanation could be selection bias, as women referred for densitometry are often those with perceived risk factors for osteoporosis.

A further limitation is that BMD was assessed only at the lumbar spine (L1–L4). This choice was determined by the availability of the densitometer in the study centre, which was calibrated and routinely used for lumbar spine assessment. Although measurements at the femoral neck or total hip are considered highly predictive of fracture risk, lumbar spine DXA remains a widely accepted and validated method for diagnosing osteoporosis. Nevertheless, this limitation should be acknowledged, as bone density at the hip is less influenced by degenerative changes and provides complementary information.

It should also be noted that we did not apply the Polish FRAX model. Not all clinical variables required for FRAX calculation were available in our dataset, which precluded its use. Future studies in Polish postmenopausal women should incorporate FRAX-based 10-year fracture probability, as this tool would greatly enhance the clinical relevance of genetic and clinical risk profiling.

Another limitation is the lack of adjustment for multiple testing. Given the number of comparisons performed, there is a risk of false positive findings. Although we used aconventional significance threshold of *p* < 0.05, future studies should consider more stringent corrections, such as Bonferroni adjustment or false discovery rate (FDR), to better control for type I error.

While the overall cohort was relatively large (N = 590), the frequency of some genotypes was low, which may have limited statistical power to detect small or moderate associations. Therefore, negative results should be interpreted with caution, and replication in larger multicentre cohorts is warranted. Finally, the study was cross-sectional, and we did not assess vitamin D, calcium intake, or physical activity, which are key determinants of bone health. Although we included variables such as BMI and smoking status in the regression analyses, many other factors that could influence the outcomes were not accounted for, including diet, hormone levels, medication use, and comorbidities. Incorporating these variables would allow a better understanding of the mechanisms that may modify genotype-related risk. Moreover, the lack of long-term follow-up prevents assessment of the impact of genotypes on changes in bone mineral density over time. Longitudinal studies could provide valuable insights into dynamic changes in BMD and their association with genetic variants, particularly in the progression of osteopenia or osteoporosis.

## 4. Materials and Methods

### 4.1. Patients

A total of 700 postmenopausal women (12 months after the last menstrual period) were invited to participate in the study during bone mineral density imaging using the DXA method in the Densitometry Laboratory of the Clinical Hospital No. 1 of the Pomeranian Medical University in Szczecin. Written consent was given by 605 (86.43%) women of Caucasian and Polish nationality to blood collection for the analysis of two polymorphic variants of the *COL1A1* and *TNFRSF11B* genes. Each patient was asked to complete a questionnaire in which she provided her clinical data (age, height, current and birth weight, age at first and last menstrual period, and number of pregnancies). Due to missing data in some questionnaires (N = 15), the results for 590 people are presented. Prior to enrolment, all participants were informed about the study’s objectives and procedures. The study protocol received approval from the Bioethics Committee of the Pomeranian Medical University in Szczecin (Approval No. KB-0012/100/15) and adhered to the principles of the Declaration of Helsinki (1975, revised in 2000). Written informed consent was obtained from all participants.

Anthropometric measurements, including body weight and height, were recorded to calculate body mass index (BMI). Bone mineral density (BMD) assessments were performed, and values were compared to both young-adult (YA) and age-matched (AM) reference standards.

The analysis included women who had been postmenopausal for at least one year and were not receiving any treatment known to affect bone mass. This included the absence of medications such as selective oestrogen receptor modulators (SERMs), calcitonin, bisphosphonates, heparin, steroids, thyroid hormones, antiepileptic drugs, GnRH analogues, tibolone, anti-resorptive agents, statins, and ACE inhibitors. Participants also had no history of hormone replacement therapy (HRT). Women with bilateral oophorectomy or those diagnosed with conditions that could influence bone loss such as endocrine or metabolic disorders, haematologic diseases, cancer, kidney disease, autoimmune conditions, and connective tissue disorders were excluded from the study.

### 4.2. Determination of Bone Mineral Density

Eligible participants underwent bone densitometry using a Lunar DPX100 device (Lunar Corp., Madison, WI, USA), with a measurement precision of 0.5%. BMD was measured at the lumbar spine (L1–L4) using dual-energy X-ray absorptiometry (DXA). The BMD values were expressed in absolute terms (g/cm^2^) and compared to age-specific reference values. T-scores and Z-scores were calculated to classify the participants. Measurements indicative of degenerative changes in the lumbar spine were excluded. Based on T-scores, the participants were divided into three groups: a control group (N = 350, T-score > −1.0), women with osteopenia (N = 105, T-score between −1.0 and −2.0), and women with osteoporosis (N = 135, T-score < −2.5). Ratios of the mean BMD to the mean for young adults (YA) and by age (age-matched, AM) were also assessed.

### 4.3. Analysis of COL1A1 and OPG Polymorphisms

The *COL1A1* polymorphisms (rs1107946, rs1800012) and *TNFRSF11B* rs2073617 polymorphism were analysed to investigate their association with the development of osteoporosis and osteopenia in postmenopausal women. Genomic DNA was extracted from peripheral blood leukocytes using the commercial Macherey-Nagel NucleoSpin^®^ Blood kit (Macherey-Nagel GmbH & Co., Duren, Germany), following the manufacturer’s instructions. Genotyping was conducted using real-time polymerase chain reaction (rt-PCR), which employs fluorescently labelled hybridisation probes that bind to specific DNA sequences. The PCR program began with an initial denaturation at 95 °C for 10 min. Each of the 45 cycles included denaturation at 95 °C for 10 s, annealing at 60 °C for 10 s, and extension at 72 °C for 15 s. The protocol concluded with a melting step, during which the temperature was increased to 95 °C to denature the amplified products.

The LightCycler^®^96 instrument (Roche Diagnostics, Mannheim, Germany) and its associated Basic Software were used for molecular analysis. Genotyping was based on fluorescence data obtained during the melting curve analysis. Specific primers and probes were used for each polymorphism, including LightSNiP reagents (TIBMolbiol, Berlin, Germany), for rs1107946, rs1800012, and rs2073617.

### 4.4. Statistical Analysis

Statistical analysis of the study results was performed in the R program (version 4.5.0, http://cran.r-project.org, 20 May 2025) [30]. The compliance of the distribution of continuous variables with the normal distribution was assessed using the Shapiro–Wilk test, and the homogeneity of variance was assessed using Levene’s test. Continuous variables arepresented using descriptive statistics (arithmetic mean with standard deviation (±SD), median with lower and upper quartiles (IQR), minimum and maximum values), and two groups were compared using the Shapiro–Wilk and Mann–Whitney tests. Correlation was analysed using the Spearman correlation coefficient. Qualitative data are presented as number (N) and percentage (%), and their comparison was performed using Pearson’s χ2 test or Fisher’s test, in case of small group size. Comparison of quantitative variable values in three groups was performed using one-way analysis of variance (Anova) or Kruskal–Wallis. After detecting statistically significant differences, Tukey’s or Dunn’s post hoc tests were used to identify statistically significant groups. The agreement of genotype distributions with the predicted distributions was analysed according to the Hardy–Weinberg law (Pearson χ2 test). To compare genotype and allele frequencies between groups, logistic regression was used and the odds ratio (OR) with a 95% confidence interval (95%CI) and *p*-value (Wald test) were calculated for four genetic models: codominant, dominant, recessive, and log-additive. The Akaike information criterion (AIC) was used to evaluate the models. Posthoc power was calculated using the “genpwr” R package (version 4.2.2) [31]. Statistical significance was set at *p* < 0.05.

## 5. Conclusions

The results of this study suggest that the rs1800012 *COL1A1* polymorphism may be a risk factor for osteopenia in postmenopausal women, particularly in interaction with environmental factors such as BMI and smoking. The rs1107946 variant did not demonstrate a significant direct effect on BMD, but may exert an indirect effect through body composition. No clear association with bone mineral density was confirmed for the rs2073617 *TNFRSF11B* variant, although the trends suggest its potential involvement in bone metabolism, warranting further research.

This study is limited by its cross-sectional nature and the lack of data on vitamin D, calcium, and physical activity—factors that significantly influence bone metabolism. The obtained data confirm the complex nature of the genetic determinants of osteoporosis and emphasise the importance of investigating gene–environment interactions in its pathogenesis.

Both prenatal factors (birth weight) and adult factors (BMI, smoking) should be considered in bone health assessment. Future multicentre longitudinal studies with larger cohorts and comprehensive risk profiling, including FRAX, are needed to better understand the complex interactions between genes, the environment, and bone health.

Moreover, future research should explore not only gene–environment interactions but also gene–gene interactions. Considering the combined effects of multiple genetic variants may provide deeper insight into the molecular mechanisms underlying osteoporosis and bone mineral density regulation.

## Figures and Tables

**Table 1:** Characteristics of the studied population (N = 590).

Analysed Variable	Mean ± SD	Median [IQR]	(Min/Max)
Age (years) <50 ≥50	54.83 ± 7.59 271 (45.93%) 319 (54.07%)	55 [50.00;60.00]	(30/78)
Birth weight (g)	3284.63 ± 504.77	3280 [3000;3600]	(1600/5100)
Current body weight (kg)	66.24 ± 11.57	65 [58.00;72.00]	(41–114)
Current height (cm)	162.19 ± 5.62	162 [158.00;165.00]	(150–180)
BMI (kg/m^2^) <25 ≥25	25.16 ± 4.18 339 (57.46%) 251 (42.54%)	24.65 [22.23;27.12]	(17.1–43.43)
Age at menarche (years) ≤12 13–15 ≥16	12.89 ± 1.97 272 (46.10%) 261 (44.24%) 57 (9.66%)	13 [11.00;14.00]	(9/17)
Age at menopause (years) ≤45 46–54 ≥55	48.26 ± 4.9 151 (25.59%) 381 (64.58%) 58 (9.83%)	48 [45.00;52.00]	(30/60)
Reproductive period (years)	35.36 ± 5.43	36 [32.00;39.00]	(17–48)
Years since menopause	6.45 ± 5.72	5 [2.00;10.00]	(1–26)
Number of pregnancies	1.91 ± 1.24	2 [1.00;3.00]	(0–7)
Smoking yes no	156 (26.44%) 434 (73.56%)		

BMI—Body mass index.

**Table 2:** Comparison of clinical data between groups of women with normal and reduced bone mineral density (BMD).

Variable	Normal BMD T-Score > −1 N = 350	Reduced BMD T-Score ≤ −1 N = 240	*p*
Age (years) <50 ≥50	54.6 ± 7.2 158 (45.1%) 192 (54.9%)	55.2 ± 8.1 113 (47.1%) 127 (52.9%)	0.293 0.704
Birth weight (g)	3410 [3260;3680]	3095 [2780;3475]	<0.001
Current body weight (kg)	68.7 ± 12.0	62.7 ± 9.9	<0.001
Current height (cm)	162.9 ± 5.8	161.1 ± 5.1	<0.001
BMI (kg/m^2^) <25 ≥25	25.9 ± 4.5 187 (53.4%) 163 (46.6%)	24.1 ± 3.4 152 (63.3%) 88 (36.7%)	<0.001 0.021
Age at menarche (years) ≤12 13–15 ≥16	13.0 [11.0;15.0] 162 (46.3%) 154 (44.0%) 34 (9.7%)	13.0 [11.0;14.0] 110 (45.8%) 107 (44.6%) 23 (9.6%)	0.466 0.990
Age at menopause (years) ≤45 46–54 ≥55	48.0 [46.0;52.0] 86 (24.6%) 223 (63.7%) 41 (11.7%)	49.0 [45.0;52.0] 65 (27.1%) 158 (65.8%) 17 (7.1%)	0.907 0.169
Reproductive period (years)	36.0 [32.0;39.0]	36.0 [32.0;39.0]	0.993
Years since menopause	5.0 [1.0;9.0]	5.0 [2.0;10.5]	0.005
Number of pregnancies	2.0 [1.0;3.0]	2.0 [1.0;3.0]	0.464
Smoking yes no	269 (76.9%) 81 (23.1%)	165 (68.8%) 75 (31.2%)	0.036

BMD—Body mass density, BMI—Body mass index.

**Table 3:** Descriptive statistics for bone mineral density parameters of the lumbar spine segments L2–L4 were obtained using DXA in the control, osteopenia, and osteoporosis groups.

Group	Variable	Mean ± SD	Median [IQR]	Min/Max
Control T-score > −1 N = 350	BMD (g/cm^2^)	1.196 ± 0.092	1.179 [1.121;1.235]	1.08/1.47
	Young-Adult (%) T-score	100.071 ± 8.092 0.011 ± 0.846	98 [94;104] −0.170 [−0.670;0.400]	90/123 −0.97/2.26
	Age-Matched (%) Z-score	107.863 ± 10.695 0.477 ± 1.015	107 [100;113] 0.530 [−0.110;1.120]	91/133 −1.85/2.65
Osteopenia T-score from −1.0 to −2.5 N = 105	BMD (g/cm^2^)	0.982 ± 0.052	0.972 [0.938;1.032]	0.90/1.07
	Young-Adult (%) T-score	81.886 ± 4.353 −1.803 ± 0.430	81 [78;86] −1.900 [−2.180;−1.440]	75/89 −2.49/−1.05
	Age-Matched (%) Z-score	89.905 ± 6.560 −0.873 ± 0.608	90 [84;94] −0.960 [−1.310;−0.410]	74/108 −2.36/0.77
Osteoporosis T-score ≤ −2.5 N = 135	BMD (g/cm^2^)	0.818 ± 0.061	0.822 [0.774;0.875]	0.63/0.90
	Young-Adult (%) T-score	68.311 ± 4.900 −3.179 ± 0.501	69 [65;73] −3.140 [−3.545;−2.720]	53/75 −4.73/−2.50
	Age-Matched (%) Z-score	78.844 ± 6.442 −1.446 ± 0.845	79 [76;81] −1.580 [−2.015;−1.160]	61/92 −3.13/0.98

BMD—Body mass density.

**Table 4:** Association analysis of variants rs1800012 and rs1107946 of the *COL1A1* gene and rs2073617 of the *TNFRSF11B* gene with reduced bone mineral density (crude model).

Genotypes/Models	Normal BMD T-Score > −1 N = 350	Reduced BMD T-Score ≤ −1 N = 240	OR (95%CI)	*p*	AIC
rs1800012 *COL1A1*					
GG	258 (73.7)	158 (65.8)	1.00	0.116	799.0
GT	83 (23.7)	75 (31.2)	1.48 (1.02–2.14)		
TT	9 (2.6)	7 (2.9)	1.27 (0.46–3.48)		
Dominant	92 (26.3)	82 (34.2)	1.46 (1.02–2.08)	0.040	797.1
Recessive	341 (97.4)	233 (97.1)	1.14 (0.42–3.10)	0.800	801.2
Log-additive	350 (59.3)	240 (40.7)	1.35 (0.99–1.84)	0.061	797.8
rs1107946 *COL1A1*					
GG	232 (66.3)	162 (67.5)	1.00	0.873	803.0
GT	108 (30.9)	70 (29.2)	0.93 (0.65–1.33)		
TT	10 (2.9)	8 (3.3)	1.15 (0.44–2.97)		
Dominant	118 (33.7)	78 (32.5)	0.95 (0.67–1.34)	0.758	801.2
Recessive	340 (97.1)	232 (96.7)	1.17 (0.46–3.02)	0.742	801.2
Log-additive	350 (59.3)	240 (40.7)	0.98 (0.72–1.32)	0.871	801.3
rs2073617 *TNFRSF11B*					
TT	105 (30.0)	67 (27.9)	1.00	0.427	801.6
TC	168 (48.0)	109 (45.4)	1.02 (0.69–1.50)		
CC	77 (22.0)	64 (26.7)	1.30 (0.83–2.05)		
Dominant	245 (70.0)	173 (72.1)	1.11 (0.77–1.59)	0.584	801.0
Recessive	273 (78.0)	176 (73.3)	1.29 (0.88–1.89)	0.193	799.6
Log-additive	350 (59.3)	240 (40.7)	1.14 (0.91–1.43)	0.268	800.1

BMD—Body mass density.

**Table 5:** Association analysis of variants rs1800012 and rs1107946 of the *COL1A1* gene and rs2073617 of the *TNFRSF11B* gene with reduced bone mineral density, N = 240 (model adjusted for BMI and smoking).

SNP	Genotypes/Models	OR (95%CI)	*p*	AIC
rs1800012 *COL1A1*	GG	1.00	0.273	769.5
	GT	1.37 (0.93–2.00)		
	TT	0.98 (0.34–2.78)		
	Dominant	1.33 (0.92–1.92)	0.136	767.9
	Recessive	0.89 (0.31–2.50)	0.818	770.0
	Log-additive	1.23 (0.89–1.70)	0.219	768.6
rs1107946 *COL1A1*	GG	1.00	0.779	771.6
	GT	0.88 (0.61–1.28)		
	TT	1.05 (0.39–2.82)		
	Dominant	0.89 (0.62–1.28)	0.541	769.7
	Recessive	1.10 (0.41–2.91)	0.852	770.0
	Log-additive	0.93 (0.68–1.27)	0.636	769.9
rs2073617 *TNFRSF11B*	TT	1.00	0.467	770.6
	TC	0.92 (0.61–1.38)		
	CC	1.20 (0.75–1.92)		
	Dominant	1.01 (0.69–1.47)	0.965	770.1
	Recessive	1.27 (0.85–1.88)	0.242	768.7
	Log-additive	1.09 (0.86–1.38)	0.470	769.6

**Table 6:** Allele analysis of the rs1800012 and rs1107946 variants of the *COL1A1* gene and rs2073617 of the *TNFRSF11B* gene in women with normal and reduced bone mineral density (BMD).

SNP	Alleles	Normal BMD T-Score > −1 N = 700	Reduced BMD T-Score ≤ −1 N = 480	OR (95%CI)	*p*
rs1800012 *COL1A1*	G	599 (85.57%)	391 (81.46%)	1.35 (0.99–1.84)	0.060
	T	101 (14.43%)	89 (18.54%)		
rs1107946 *COL1A1*	G	572 (81.71%)	394 (82.08%)	0.98 (0.72–1.32)	0.872
	T	128 (18.29%)	86 (17.92%)		
rs2073617 *TNFRSF11B*	T	378 (54.00%)	243 (50.62%)	1.14 (0.91.1.44)	0.254
	C	322 (46.00%)	237 (49.38%)		

**Table 7:** Association analysis of rs1800012 and rs1107946 variants in groups of women with osteoporosis and control group.

Genotypes/Models	Crude Model			Model Adjusted for BMI and Smoking		
	OR (95%CI)	*p*	AIC	OR (95%CI)	*p*	AIC
rs1800012 *COL1A1*						
GG	1.00	0.213	576.6	1.00	0.220	538.6
GT	1.43 (0.92–2.23)			1.35 (0.85–2.15)		
TT	0.63 (0.13–2.97)			0.45 (0.09–2.20)		
Dominant	1.36 (0.88–2.09)	0.170	575.8	1.25 (0.79–1.97)	0.338	538.7
Recessive	0.57 (0.12–2.67)	0.451	577.1	0.41 (0.08–1.99)	0.232	538.2
Log-additive	1.22 (0.83–1.79)	0.314	576.6	1.11 (0.74–1.66)	0.619	539.4
rs1107946 *COL1A1*						
GG	1.00	0.829	579.3	1.00	0.611	540.6
GT	0.90 (0.58–1.4)			0.81 (0.51–1.28)		
TT	0.75 (0.2–2.78)			0.71 (0.18–2.79)		
Dominant	0.89 (0.58–1.36)	0.584	577.3	0.80 (0.51–1.25)	0.330	538.7
Recessive	0.77 (0.21–2.85)	0.693	577.5	0.75 (0.19–2.95)	0.680	539.5
Log-additive	0.89 (0.61–1.3)	0.546	577.3	0.82 (0.55–1.22)	0.323	538.6
rs2073617 *TNFRSF11B*						
TT	1.00	0.084	574.7	1.00	0.155	537.9
TC	1.42 (0.86–2.35)			1.30 (0.77–2.19)		
CC	1.88 (1.07–3.30)			1.78 (0.99–3.21)		
Dominant	1.57 (0.98–2.51)	0.056	574.0	1.45 (0.88–2.37)	0.138	537.4
Recessive	1.49 (0.95–2.34)	0.083	574.6	1.49 (0.93–2.39)	0.096	536.9
Log-additive	1.37 (1.04–1.81)	0.026	572.7	1.34 (0.99–1.80)	0.054	535.9

BMI—Body mass index.

## Data Availability

The original contributions presented in this study are included in the article. Further inquiries can be directed to the corresponding author.

## Supplementary Materials

The following supporting information can be downloaded at: https://www.mdpi.com/article/10.3390/ijms26188894/s1.
