# Supplementary material for: The Influence of Clinical Factors and Genetic Variants of COL1A1 and TNFRSF11B on Bone Mineral Density in Postmenopausal Women
Source: Int J Mol Sci. 2025 Sep 12;26(18):8894. doi: 10.3390/ijms26188894 (PMC12470021; doi:10.3390/ijms26188894)
Supplement: Supplementary file 1 [file ijms-26-08894-s001.zip › ijms-3845532-supplementary.pdf]

International Journal of *Molecular Sciences*

MDPI

Supplementary Materials

The influence of clinical factors and genetic variants of *COL1A1* and *TNFRSF11B* on bone mineral density in postmenopausal women

Katarzyna Kotrych[1,†], Maciej Wojtuń[1†], Aleksandra Górska[2], Anna Bogacz[3,*], Michał Soczawa[4],Izabela Uzar[5], Jarosław Gorący[6], Maciej Brązert[7],Bogusław Czerny[2,5] and Adam Kamiński[8]

1. Department of General and Dental Radiology, Pomeranian Medical University in Szczecin, al. Powstańców Wielkopolskch 72, 70-111 Szczecin, Poland; kotrych1@gmail.com (K.K.); maciej.wojtun@pum.edu.pl (M.W.)
2. Department of Stem Cells and Regenerative Medicine, Institute of Natural Fibres and Medicinal Plants, Kolejowa 2, 62-064 Plewiska, Poland; aleksandra.gorska@iwnirz.pl (A.G.)
3. Chair of Medical Biotechnology, Department of Cancer Immunology, Poznan University of Medical Sciences, Rokietnicka8, Poznan 60-806, Poland;aniabogacz23@o2.pl (A.B.)
4. Department and Clinic of Urology and Urological Oncology, Pomeranian Medical University in Szczecin, al. Powstańców Wielkopolskich 72, 70-111 Szczecin, Poland; michal.soczawa@pum.edu.pl (M.S.)
5. Department of Pharmacology and Pharmacoeconomics, Pomeranian Medical University in Szczecin, 71-230 Szczecin, Poland; uzari@wp.pl (I.U.), bczerny@wp.pl (B.C.)
6. Independent Laboratory of Invasive Cardiology, Pomeranian Medical University, 70-111 Szczecin, Poland; jaroslaw.goracy@pum.edu.pl (J.G.)
7. Department of Infertility Diagnostics and Treatment, Poznan University of Medical Sciences, Polna 33, 60-535 Poznan, Poland; maciejbrazert@ump.edu.pl (M.B.)
8. Department of of Children Orthopedics and Musculosceletal Oncology, Pomeranian Medical University in Szczecin, UniiLubelskiej 1, 71-252 Szczecin,, Poland; emluc@wp.pl (A.K.)

* Correspondence: aniabogacz23@o2.pl

† These authors contributed equally to this work.

Citation: Kotrych,K.; Wojtuń, M.; Górska, A.; Bogacz, A.; Soczawa, M.; Uzar, I.; Gorący, J.; Brązert, M.; Czerny, B.; Kamiński, A.. *Int. J. Mol. Sci.* **2025**, *26*, x. https://doi.org/10.3390/xxxxx

Academic Editor(s): Name

Received: date
Revised: date
Accepted: date
Published: date

Copyright: © 2025 by the authors. Submitted for possible open access publication under the terms and conditions of the Creative Commons Attribution (CC BY) license (https://creativecommons.org/licenses/by/4.0/).

Table S1. Association analysis of the rs1800012, rs1107946 and rs2073617 variants in groups of women with osteopenia and the control group.

Genotypes/models	Crude model			Model adjusted for BMI and smoking		
	OR (95%CI)	p	AIC	OR (95%CI)	p	AIC
**rs1800012 *COL1A1***						
GG	1.00	0.131	493.5	1.00	0.275	491.8
GT	1.53 (0.94-2.49)			1.43 (0.87-2.33)		
TT	2.14 (0.69-6.59)			1.78 (0.57-5.63)		
Dominant	1.59 (1.00-2.53)	0.052	491.8	1.46 (0.91-2.35)	0.118	489.9
Recessive	1.89 (0.62-5.78)	0.278	494.4	1.59 (0.51-4.95)	0.436	491.8
Log-additive	1.50 (1.02-2.22)	0.044	491.5	1.39 (0.93-2.07)	0.111	489.8
**rs1107946 *COL1A1***						
GG	1.00	0.648	496.7	1.00	0.688	493.6
GT	0.97 (0.60-1.56)			0.95 (0.59-1.55)		
TT	1.68 (0.56-5.08)			1.61 (0.52-4.93)		
Dominant	1.03 (0.65-1.62)	0.914	495.6	1.01 (0.64-1.61)	0.966	492.4
Recessive	1.70 (0.57-5.09)	0.358	494.7	1.63 (0.54-4.96)	0.399	491.7
Log-additive	1.09 (0.73-1.61)	0.686	495.4	1.07 (0.72-1.59)	0.747	492.3
**rs2073617 *TNFRSF11B***						
TT	1.00	0.393	495.7	1.00	0.277	491.8
TC	0.71 (0.43 - 1.17)			0.66 (0.40 - 1.10)		
CC	0.86 (0.48 - 1.55)			0.82 (0.45 - 1.49)		
Dominant	0.76 (0.48 - 1.20)	0.235	494.2	0.71 (0.44 - 1.13)	0.155	490.4
Recessive	1.05 (0.62 - 1.77)	0.853	495.6	1.05 (0.62 - 1.77)	0.869	492.4
Log-additive	0.90 (0.67 - 1.22)	0.509	495.1	0.88 (0.65 - 1.20)	0.411	491.7

Table S2. Comparison of clinical data of patients divided according to genotypes of the rs1800012 variant of the *COL1A1* gene.

Variable	Genotypes rs1800012			p
	GG (N=416)	GT (N=158)	TT (N=16)	
Age (years)	54.7 ± 7.8	55.1 ± 7.1	55.2 ± 6.7	0.880
<50	195 (46.9%)	68 (43.0%)	8 (50.0%)	0.674
≥50	221 (53.1%)	90 (57.0%)	8 (50.0%)	
Birth weight (g)	3280 [2935;3635]	3300 [3080;3500]	3340 [3020;3680]	0.826
Current body weight (kg)	67.3 ± 11.6	64.1 ± 11.1	60.8 ± 12.6	0.002
Current height (cm)	162.2 ± 5.7	162.1 ± 5.4	163.1 ± 4.9	0.798
BMI (kg/m2)	25.5 ± 4.2	24.4 ± 4.0	22.9 ± 4.5	0.001
<25	220 (52.9%)	105 (66.5%)	14 (87.5%)	0.001
≥25	196 (47.1%)	53 (33.5%)	2 (12.5%)	
Age at menarche (years)	13.0 [11.0;14.0]	13.0 [11.0;15.0]	12.5 [11.5;13.5]	
≤12	192 (46.2%)	72 (45.6%)	8 (50.0%)	0.628
13-15	186 (44.7%)	67 (42.4%)	8 (50.0%)	0.574
≥16	38 (9.1%)	19 (12.0%)	0 (0.0%)	
Age at menopause (years)	49.0 [45.0;52.0]	48.0 [46.0;51.0]	49.0 [46.5;52.0]	
≤45	109 (26.2%)	39 (24.7%)	3 (18.8%)	0.688
46-54	262 (63.0%)	108 (68.4%)	11 (68.8%)	0.590
≥55	45 (10.8%)	11 (7.0%)	2 (12.5%)	
Reproductive period (years)	36.0 [32.0;39.0]	36.0 [31.0;38.0]	36.5 [33.5;40.5]	0.498
Years since menopause	5.0 [2.0;10.0]	5.0 [2.0;10.0]	4.0 [1.5; 9.5]	0.638
Number of pregnancies	2.0 [1.0; 2.0]	2.0 [1.0; 3.0]	2.0 [1.0; 3.0]	0.879
Smoking				
yes	298 (71.6%)	123 (77.8%)	13 (81.2%)	0.250
no	118 (28.4%)	35 (22.2%)	3 (18.8%)	

Table S3. Mean body weight and BMI of women in the control, osteopenia and osteoporosis groups divided according to genotypes of the rs1800012 variant of the *COL1A1* gene.

Group	Variable	Genotypes rs1800012			p
		GG	GT	TT	
**Normal BMD T-score > -1 N=350**	Current body weight (kg)	69.4 ± 11.9	67.0 ± 12.0	62.7 ± 15.2	0.086
	BMI (kg/m2)	26.2 ± 4.5	25.2 ± 4.4	23.1 ± 5.5	0.043
	<25	126 (48.8%)	53 (63.9%)	8 (88.9%)	0.006
	≥25	132 (51.2%)	30 (36.1%)	1 (11.1%)	
**Osteopenia T-score from -1.0 to -2.5 N=105**	Current body weight(kg)	67.5 ± 11.3	62.1 ± 9.7	58.0 ± 10.3	0.021
	BMI (kg/m2)	25.4 ± 4.0	23.6 ± 3.7	21.8 ± 3.6	0.027
	<25	28 (41.8%)	20 (60.6%)	4 (80.0%)	0.079
	≥25	39 (58.2%)	13 (39.4%)	1 (20.0%)	
**Osteoporosis T-score ≤ -2.5 N=135**	Current body weight(kg)	61.1 ± 8.2	59.8 ± 8.6	59.5 ± 4.9	0.708
	BMI (kg/m2)	23.7 ± 2.6	23.3 ± 2.9	24.2 ± 0.9	0.666
	<25	66 (72.5%)	32 (76.2%)	2 (100.0%)	0.634
	≥25	25 (27.5%)	10 (23.8%)	0 (0.0%)	

Table S4. Results of bone mineral density of the lumbar spine segments L2–L4 was measured by DXA and compared between genotypes of the rs1800012 variant of the *COL1A1* gene.

Variable	GG (N=416)	GT (N=158)	TT (N=16)	p
BMD (g/cm^2)	1.1 [0.9; 1.2]	1.1 [0.9; 1.2]	1.1 [1.0; 1.2]	0.270
Young-Adult (%)	93.0 [77.0;101.0]	90.0 [75.0;98.0]	92.5 [79.5;99.0]	0.211
T-score	-0.8 [-2.3; 0.1]	-0.9 [-2.5;-0.2]	-0.8 [-1.9;-0.1]	0.229
Age-Matched (%)	98.0 [86.0;108.0]	95.5 [84.0;107.0]	100.0 [91.0;102.5]	0.271
Z-score	-0.2 [-1.1; 0.7]	-0.5 [-1.5; 0.6]	0.0 [-0.8; 0.4]	0.246

Table S5. Comparison of clinical data of patients divided according to genotypes of the rs1107946 variant of the *COL1A1* gene.

Variable	Genotypes rs1107946			p
	GG (N=394)	GT (N=178)	TT (N=18)	
Age (years)	54.9 ± 7.5	54.8 ± 7.8	53.9 ± 6.6	0.873
<50	181 (45.9%)	80 (44.9%)	10 (55.6%)	0.690
≥50	213 (54.1%)	98 (55.1%)	8 (44.4%)	
Birth weight (g)	3300 [3000;3600]	3260 [2950;3600]	3270 [3000;3700]	0.419
Current body weight(kg)	66.7 ± 11.8	65.6 ± 11.0	62.3 ± 12.6	0.195
Current height (cm)	162.3 ± 5.7	162.1 ± 5.6	160.7 ± 5.6	0.503
BMI (kg/m2)	25.3 ± 4.2	25.0 ± 4.1	24.2 ± 4.9	0.479
<25	224 (56.9%)	102 (57.3%)	13 (72.2%)	0.435
≥25	170 (43.1%)	76 (42.7%)	5 (27.8%)	
Age at menarche (years)	13.0 [11.0;14.0]	13.0 [11.0;14.0]	12.0 [12.0;14.0]	
≤12	174 (44.2%)	87 (48.9%)	11 (61.1%)	0.867
13-15	186 (47.2%)	70 (39.3%)	5 (27.8%)	0.225
≥16	34 (8.6%)	21 (11.8%)	2 (11.1%)	
Age at menopause (years)	49.0 [45.0;52.0]	48.0 [45.0;50.0]	49.0 [46.0;52.0]	
≤45	99 (25.1%)	50 (28.1%)	2 (11.1%)	0.181
46-54	252 (64.0%)	116 (65.2%)	13 (72.2%)	0.268
≥55	43 (10.9%)	12 (6.7%)	3 (16.7%)	
Reproductive period (years)	36.0 [32.0;40.0]	35.5 [31.0;38.0]	35.5 [33.0;40.0]	0.250
Years since menopause	5.0 [2.0;10.0]	5.0 [2.0;10.0]	4.0 [2.0; 7.0]	0.763
Number of pregnancies	2.0 [1.0; 3.0]	2.0 [1.0; 2.0]	2.0 [2.0; 2.0]	0.748
Smoking				
yes	293 (74.4%)	126 (70.8%)	15 (83.3%)	0.423
no	101 (25.6%)	52 (29.2%)	3 (16.7%)	

Table S6. Results of bone mineral density of the lumbar spine segments L2–L4 was measured by DXA and compared between genotypes of the rs1107946 variant of the *COL1A1* gene.

Variable	Genotypesrs1107946			p
	GG (N=394)	GT (N=178)	TT (N=18)	
BMD (g/cm²)	1.1 [0.9; 1.2]	1.1 [0.9; 1.2]	1.1 [1.0; 1.2]	0.859
Young-Adult (%)	91.0 [76.0;100.0]	93.0 [77.0;100.0]	90.0 [80.0;100.0]	0.813
T-score	-0.9 [-2.4; 0.1]	-0.7 [-2.3; 0.1]	-1.0 [-2.0; 0.0]	0.832
Age-Matched (%)	98.0 [84.0;108.0]	100.0 [88.0;109.0]	96.0 [87.0;105.0]	0.491
Z-score	-0.3 [-1.3; 0.7]	-0.1 [-1.2; 0.6]	-0.3 [-1.4; 0.4]	0.659

Table S7. Comparison of clinical data of patients divided according to genotypes of the rs2073617 variant of the *TNFRSF11B* gene.

Variable	Genotypesrs2073617			p
	TT (N=172)	TC (N=277)	CC (N=141)	
Age (years)<50<br>≥50	55.4 ± 7.3<br>73 (42.4%)<br>99 (57.6%)	54.1 ± 7.5<br>137 (49.5%)<br>140 (50.5%)	55.6 ± 8.0<br>61 (43.3%)<br>80 (56.7%)	0.089<br>0.268
Birth weight (g)	3260 [2910;3510]	3300 [3000;3600]	3360 [3110;3680]	0.009
Current body weight (kg)	68.0 ± 11.7	65.8 ± 11.3	65.0 ± 11.8	0.053
Current height (cm)	162.1 ± 5.4	162.4 ± 5.8	161.8 ± 5.4	0.533
BMI (kg/m2)<br><25<br>≥25	25.8 ± 4.3<br>93 (54.1%)<br>79 (45.9%)	24.9 ± 4.1<br>158 (57.0%)<br>119 (43.0%)	24.8 ± 4.1<br>88 (62.4%)<br>53 (37.6%)	0.047<br>0.326
Age at menarche (years)<br>≤12<br>13-15<br>≥16	13.0 [11.0;14.0]<br>85 (49.4%)<br>68 (39.5%)<br>19 (11.0%)	13.0 [12.0;14.0]<br>112 (40.4%)<br>134 (48.4%)<br>31 (11.2%)	12.0 [11.0;14.0]<br>73 (51.8%)<br>60 (42.6%)<br>8 (5.7%)	0.070<br>0.072
Age at menopause (years)≤45<br>46-54<br>≥55	50.0 [45.0;52.0]<br>42 (24.4%)<br>112 (65.1%)<br>18 (10.5%)	48.0 [45.0;51.0]<br>78 (28.2%)<br>174 (62.8%)<br>25 (9.0%)	48.0 [46.0;52.0]<br>32 (22.7%)<br>96 (68.1%)<br>13 (9.2%)	0.104<br>0.753
Reproductive period (years)	36.0 [33.0;40.0]	35.0 [31.0;38.5]	36.0 [32.0;39.0]	0.076
Number of years since menopause	5.0 [2.0;10.0]	5.0 [2.0; 9.0]	5.0 [2.0;10.0]	0.508
Number of pregnancies	2.0 [1.0; 3.0]	2.0 [1.0; 3.0]	2.0 [1.0; 2.0]	0.627
Smoking<br>yes<br>no	 121 (70.3%)<br>51 (29.7%)	 203 (73.3%)<br>74 (26.7%)	 110 (78.0%)<br>31 (22.0%)	0.307

Table S8. Median birth weight (g) of patients in the control, osteopenia and osteoporosis groups divided by genotypes of the rs2073617 variant of the *TNFRSF11B* gene.

Group	Genotypesrs2073617			p
	TT	TC	CC	
**Normal BMD**<br>**T-score > -1 (N=350)**	3320 [3200;3680]	3400 [3260;3680]	3440 [3280;3800]	0.023
**Osteopenia**<br>**T-score from -1.0 to -2.5 (N=105)**	2885 [2520;3300]	3000 [2800;3320]	3375 [3095;3535]	0.013
**Osteoporosis**<br>**T-score ≤ -2.5 (N=135)**	3000 [2520;3095]	3095 [2670;3500]	3097 [2810;3260]	0.080

Table S9. Results of bone mineral density of the lumbar spine segments L2–L4 was measured by DXA and compared between genotypes of the *TNFRSF11B* gene.

**Variable**	**Genotypesrs2073617**			**p**
	**TT (N=172)**	**TC (N=277)**	**CC (N=141)**	
BMD (g/cm^2)	1.1 [1.0; 1.2]	1.1 [0.9; 1.2]	1.1 [0.9; 1.2]	0.100
Young-Adult (%)	94.0 [80.0;101.5]	93.0 [76.0;101.0]	91.0 [74.0;98.0]	0.081
T-score	-0.8 [-1.9; 0.2]	-0.9 [-2.4; 0.1]	-0.9 [-2.6;-0.2]	0.091
Age-Matched (%)	99.0 [88.0;109.0]	98.0 [85.0;108.0]	96.0 [85.0;107.0]	0.247
Z-score	-0.2 [-1.0; 0.8]	-0.2 [-1.3; 0.6]	-0.3 [-1.5; 0.5]	0.160
